# Receptor for Advanced Glycation End Products and its Inflammatory Ligands are Upregulated in Amyotrophic Lateral Sclerosis

**DOI:** 10.3389/fncel.2015.00485

**Published:** 2015-12-22

**Authors:** Judyta K. Juranek, Gurdip K. Daffu, Joanna Wojtkiewicz, David Lacomis, Julia Kofler, Ann Marie Schmidt

**Affiliations:** ^1^Department of Surgery, Columbia University Medical CenterNew York, NY, USA; ^2^Department of Medicine, New York University Langone Medical Center – New York University School of MedicineNew York, NY, USA; ^3^Department of Pathophysiology, University of Warmia and MazuryOlsztyn, Poland; ^4^Department of Neurology, University of PittsburghPittsburgh, PA, USA; ^5^Department of Pathology, University of PittsburghPittsburgh, PA, USA

**Keywords:** amyotrophic lateral sclerosis, spinal cord, RAGE, CML, S100B, HMGB1

## Abstract

Amyotrophic lateral sclerosis (ALS) is a fatal motor neuron disorder of largely unknown pathogenesis. Recent studies suggest that enhanced oxidative stress and neuroinflammation contribute to the progression of the disease. Mounting evidence implicates the receptor for advanced glycation end-products (RAGE) as a significant contributor to the pathogenesis of certain neurodegenerative diseases and chronic conditions. It is hypothesized that detrimental actions of RAGE are triggered upon binding to its ligands, such as AGEs (advanced glycation end products), S100/calgranulin family members, and High Mobility Group Box-1 (HMGB1) proteins. Here, we examined the expression of RAGE and its ligands in human ALS spinal cord. Tissue samples from age-matched human control and ALS spinal cords were tested for the expression of RAGE, carboxymethyllysine (CML) AGE, S100B, and HMGB1, and intensity of the immunofluorescent and immunoblotting signals was assessed. We found that the expression of both RAGE and its ligands was significantly increased in the spinal cords of ALS patients versus age-matched control subjects. Our study is the first report describing co-expression of both RAGE and its ligands in human ALS spinal cords. These findings suggest that further probing of RAGE as a mechanism of neurodegeneration in human ALS is rational.

## Introduction

Amyotrophic lateral sclerosis is a fatal neurodegenerative disorder of upper and lower motor neurons manifested by progressive muscle wasting, muscular atrophy, and subsequent respiratory complications, leading to paralysis of respiratory muscles and the diaphragm, with death usually occurring within 3–5 years after diagnosis (Rowland and Shneider, 2001). The pathogenesis of the disease is largely unknown; however, growing evidence suggests that the progressive neurodegeneration of motor neurons in ALS results from a combination of multiple contributing factors, such as oxidative stress, neuroinflammation, glutamate toxicity, mitochondrial dysfunction, neurofilament disorganization, impaired axonal transport, and excessive neuronal apoptosis (Mitchell and Borasio, 2007; Brites and Vaz, 2014). Despite extensive efforts, therapies to extend survival and delay the loss of neuronal function in affected subjects have not been successful.

The RAGE belongs to the family of the cell surface immunoglobulins and plays an important role in inflammation, oxidative stress, and cellular dysfunction in a number of conditions and diseases (Schmidt et al., 2000; Bierhaus et al., 2004; Daffu et al., 2013). Over the last decade, RAGE has gained attention as a potential contributor to the pathogenesis of various neurodegenerative diseases and conditions, such as Alzheimer’s disease (AD), Parkinson’s disease, Huntington’s disease, Creutzfeldt-Jakob disease, diabetic neuropathy, familial amyloid polyneuropathy, Charcot neuroarthropathy, and vasculitic neuropathy (Juranek et al., 2015).

While the specific molecular mechanisms by which RAGE contributes to neurodegeneration remain elusive, studies indicate that the detrimental actions of RAGE are triggered upon its binding to certain ligands, such as AGEs, members of the S100/calgranulin family and HMGB1 proteins (Casula et al., 2011). These ligands are implicated in inflammation and oxidative stress and subsequent activation of downstream regulatory pathways, such as NF-κB, JNK and STAT1 (Saleh et al., 2013).

In the central nervous system (CNS), RAGE expression has been noted in neurons, vascular cells, microglia, and astrocytes, suggesting its key role in the CNS (Ding and Keller, 2005). The action of RAGE in these cells depends on the specific RAGE isoform and its concentration levels on the cell surface (Ding and Keller, 2005). It has been observed that in neurons, RAGE-AGE or RAGE-Amyloid β interaction results in increased oxidative stress and NF-κB cellular activation (Yan et al., 1996; Mattson and Camandola, 2001; Mattson, 2004), while in inflammatory cells, RAGE-S100 binding triggers immune cell migration and synthesis of proinflammatory mediators (Hofmann et al., 1999). On the contrary, studies showed that in neuroblastoma cells, members of the S100 family triggered RAGE and HMGB1 activation, stimulating axonal outgrowth and improved neuronal survival (Huttunen et al., 2000; Donato, 2001). In addition, studies using mouse models of neurodegenerative disease such as diabetic peripheral neuropathy, AD and Parkinson’s disease have shown that pharmacological treatment or genetic blockade of RAGE attenuates diabetes-associated complications, plaque formation, or MPTP-induced toxicity of dopaminergic neurons, respectively (Bierhaus et al., 2004; Cho et al., 2009; Teismann et al., 2012).

Recently, we showed in diabetic mice that genetic deletion of RAGE improved post injury sciatic nerve regeneration by reducing tissue inflammation at the injury site (Juranek et al., 2013b). Other studies investigating levels of soluble RAGE (sRAGE), a natural competitor of RAGE, in ALS and AD showed a correlation between low levels of plasma sRAGE and the clinical and pathophysiological manifestation of these diseases (Emanuele et al., 2005; Ilzecka, 2009; Liang et al., 2013). In another study, human post-mortem brains of patients affected by AD and diabetes showed significantly increased RAGE and AGE levels by immunohistochemical staining (Valente et al., 2010).

Here, we examined the expression of RAGE and its ligands in the spinal cord tissue of human ALS subjects. Our findings revealed significantly increased expression of RAGE and its three ligand families in ALS as compared to age-matched controls. These data suggest that the RAGE axis may contribute to the pathogenesis of the disease, and that its blockade may be a rational target for therapeutic intervention in ALS.

## Materials and Methods

### Tissue Collection

For immunohistochemistry experiments, the study utilized de-identified age-matched control and ALS surplus thoracic spinal cord tissue selected from the Columbia University Medical Center tissue bank from deceased sporadic ALS patients (both genders; age range 63.6 ± 4.7 years), admitted previously to the Columbia University Medical Center for diagnosis and treatment, and from deceased control subjects with no neurodegenerative disease diagnosis (both genders; age range 50–70 years). The study was approved by the Institutional Review Board and all study subjects granted their written informed consent. For mRNA analysis and Western blotting, de-identified age-matched control and ALS thoracic spinal cord tissues were obtained from the University of Pittsburgh brain tissue bank for neurodegenerative diseases from deceased sporadic ALS patients and from deceased control subjects with no neurodegenerative disease diagnosis [all males with age range: ALS (58.3 ± 5.3 years), controls (62.3 ± 6.9 years); duration of disease: ALS (32 ± 8 months)]. The brain bank is approved by the University of Pittsburgh Committee for Oversight of Research Involving the Dead (CORID). Permission for research was granted by the next of kin as part of the autopsy authorization.

### Immunohistochemistry

Thoracic (T1–T3) spinal cord samples were deparaffinized and subsequently immunostained exactly as we have previously reported (Juranek et al., 2013a). Briefly, frozen samples were cut transversely at 10 μm thickness on cryostat (Microm HM 550, Thermo Scientific, Waltham, MA, USA) and collected on polylysine coated slides (SuperFrost Plus, Fisher Scientific, Pittsburgh, PA, USA), always in the same order – two control and two ALS sections per each slide set. The investigator was blinded to the coding of the sections. After collection, sections were allowed to dry for 2 h at room temperature and processed according to standard immunostaining protocol. In brief, dried sections were incubated with blocking solution (Cas-block, Invitrogen, Carlsbad, CA, USA) for 1 h and incubated overnight with following primary antibodies: goat anti-RAGE (1:100; GeneTex, Irvine, CA, USA), mouse anti-S100B (1:100, Sigma, St. Louis, MO, USA), rabbit anti-HMGB1 (1:100, Abcam, Cambridge, MA, USA), and rabbit anti-CML (1:100 Abcam). The following day, sections were rinsed 4 × 5 min in PBS, incubated with secondary antibodies: chicken anti-goat Alexa 568, chicken anti-mouse Alexa 488 and chicken anti-rabbit Alexa 633 (1:300, 1:200, and 1:200, respectively, Invitrogen, Grand Island, NY, USA) for 1 h, rinsed again 4 × 5 min in PBS and mounted in Vectashield fluorescent mounting medium with DAPI (Vector Laboratories, USA). To control specificity of secondary antibodies and minimize risk of false positive results, standard immunostaining procedure with omission or replacement of primary antibodies on sections from each tissue sample set was carried out parallel to the experimental staining. Mounted sections were allowed to stabilize for 30 min and then examined with Leica SP5 scanning confocal microscope (Leica SP5, Goettingen, Germany) at 20× objectives (Leica, Plan-Apochromat air objectives) at one focal plane. Image acquisition parameters were kept identical for each studied specimen. Following confocal image acquisition, immunofluorescent signal quantification was carried out using Image J software as previously described (Juranek et al., 2013a), according to NIH recommended guidelines and standardized procedures (Prasad and Prabhu, 2012). Quantitative analysis of the gray matter thoracic ventral cord lamina IX region immunofluorescent signal were performed on selected region of interests (ROI, 200 μm^2^), three ROIs per each studied control/ALS sample, five slides per subject.

### RNA Isolation and Quantitative RT-PCR

Total RNA was extracted from spinal cord samples using the RNeasy mini kit (Qiagen, Valencia, CA, USA). cDNA was synthesized with iScript cDNA Synthesis Kit (BioRad, Hercules, CA, USA). Quantitative RT-PCR for *AGER* was performed using the TaqMan Fast Universal Master Mix 2X with a premade primer set (Hs00542584_g1; Life Technologies, Carlsbad, CA, USA) for measurement of the indicated transcripts. The relative abundance of transcripts was normalized according to the expression of *IPO8* house keeping gene.

### Western Blot Analysis

Spinal cord tissue samples were homogenized using cell lysis buffer (Cell Signaling, Danvers, MA, USA) and the Bullet Blender (Next Advance, Averill Park, NY, USA). Total protein concentrations were quantified using Quick Start Bradford 1x Dye (Bio-Rad). Tissue homogenates (30 μg) were subjected to electrophoresis using 4–12% SDS-PAGE gels and specific protein signals were detected using the following antibodies: mouse anti-β-actin (A1978, 1:4000, Sigma); rabbit anti-RAGE (GTX23611, 1:1000, GeneTex); rabbit anti-S100B (ab52642, 1:5000, Abcam); and rabbit anti-HMGB1 (GTX101277, 1:1000, GeneTex). After incubation with indicated antibodies, protein signals were visualized with IRDye 680RD Goat anti-mouse (1:25,000; LI-COR, Lincoln, NE, USA) and IRDye 800CW Goat anti-rabbit (1:10,000; LI-COR) and protein signals were visualized using the Odyssey Infrared Imaging System Model 9120 (LI-COR). Quantification was carried out using Image J open source software as described previously (Juranek et al., 2013a).

### Statistical Analysis

All values are presented as mean ± standard error (SEM). The statistical significance of differences (*p* < 0.05) was evaluated by non-parametric two-tailed *t*-test (GraphPad Instat, La Jolla, CA, USA).

## Results

### Increased Expression of RAGE and RAGE Ligands in the ALS Spinal Cord

**FIGURE 1:**
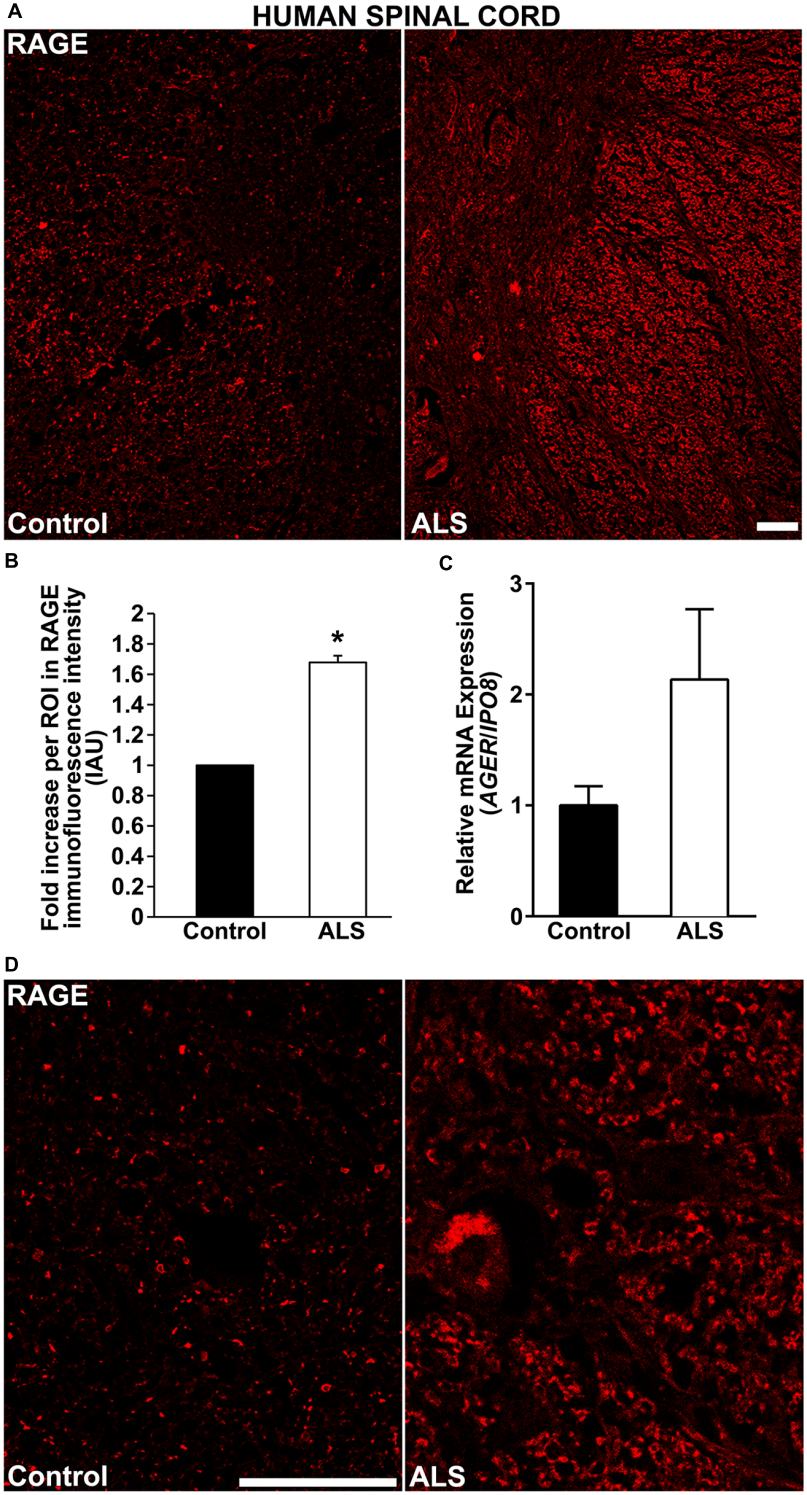
**RAGE expression is increased in human ALS spinal cord. (A)** Thoracic spinal cord sections were immunostained for RAGE expression from control (left) or ALS (right) subjects. **(B)** Quantification of RAGE immunofluorescent staining intensity in spinal cord tissue. Representative images from each group are shown; control (*n* = 6) vs. ALS samples (*n* = 5). **(C)** mRNA expression levels for *AGER* were determined by quantitative RT-PCR (*n* = 3 samples/group). **(D)** Magnified images of RAGE immunostaining in ALS and control thoracic spinal cord, motor ventral horn. Clear staining of motor neuron and surrounding area is visible in the ALS sample as compared to control. Error bars represent mean ± SEM, ^∗^*p* < 0.05. Scale bar: 100 μm.

**FIGURE 2:**
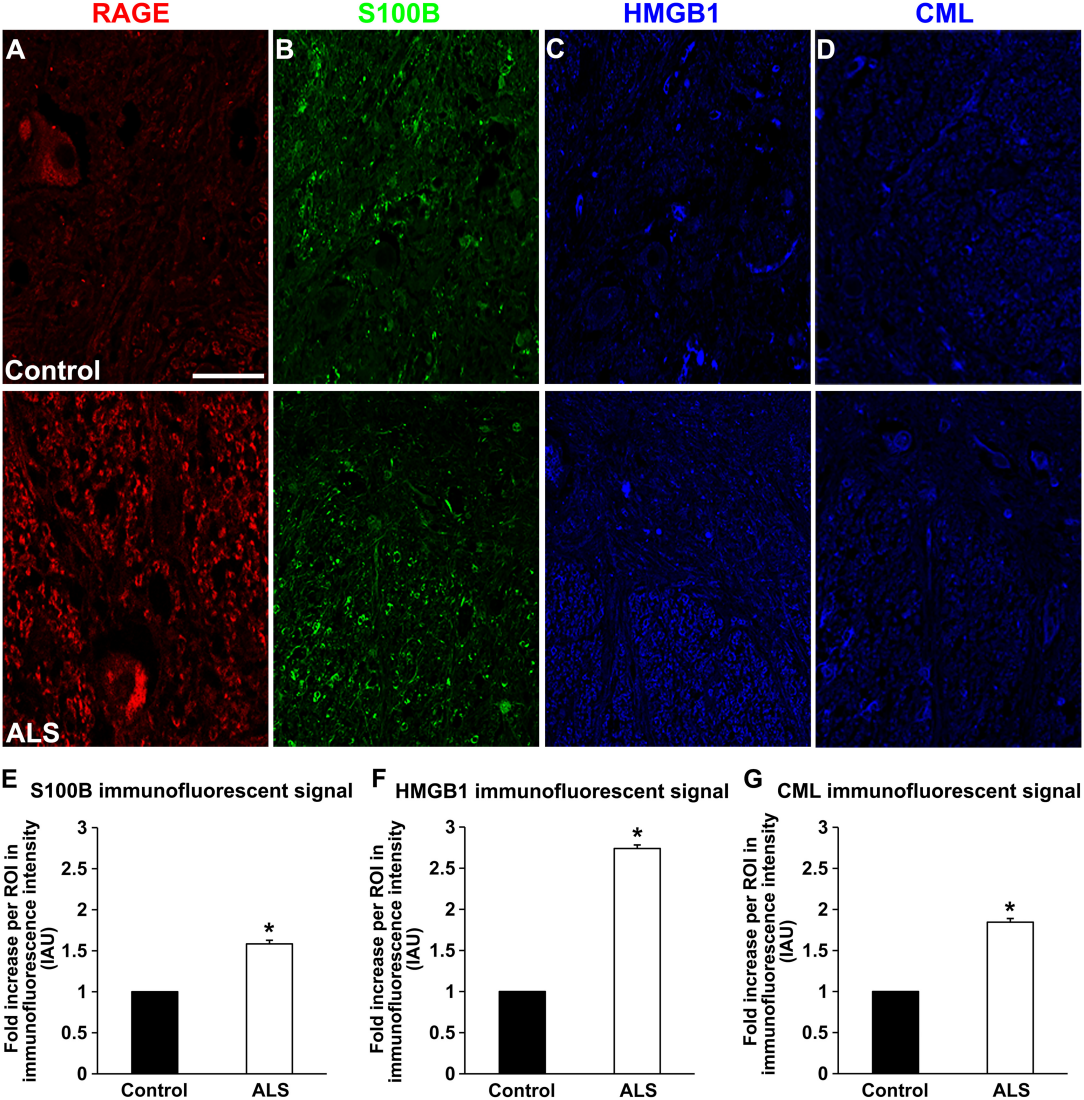
**Expression of RAGE and its ligands in control and ALS thoracic spinal cord tissue. (A)** RAGE expression in control (**A**, top), and in ALS tissue (**A**, bottom). **(B)** S100B immunostaining in control tissue (**B**, top) and in ALS tissue (**B**, bottom). **(C)** HMGB1 immunostaining in the control tissue (**C**, top) and in ALS tissue (**C**, bottom). **(D)** CML immunostaining in control spinal cord (**D**, top) and in ALS spinal cord (**D**, bottom). **(E–G)** Quantification of immunostaining intensity revealed that expression of all studied proteins was significantly increased in ALS thoracic spinal cord tissue compared to controls. S100B **(E)** was increased about 70%, HMGB1 **(F)** displayed almost threefold increase and CML **(G)** showed almost double level of increase in immunostaining between ALS and control subjects. Sections are representative of *n* = 6 control and *n* = 5 ALS tissue samples per condition. Error bars represent mean ± SEM, ^∗^*p* < 0.05. Scale bar: 50 μm.

**FIGURE 3:**
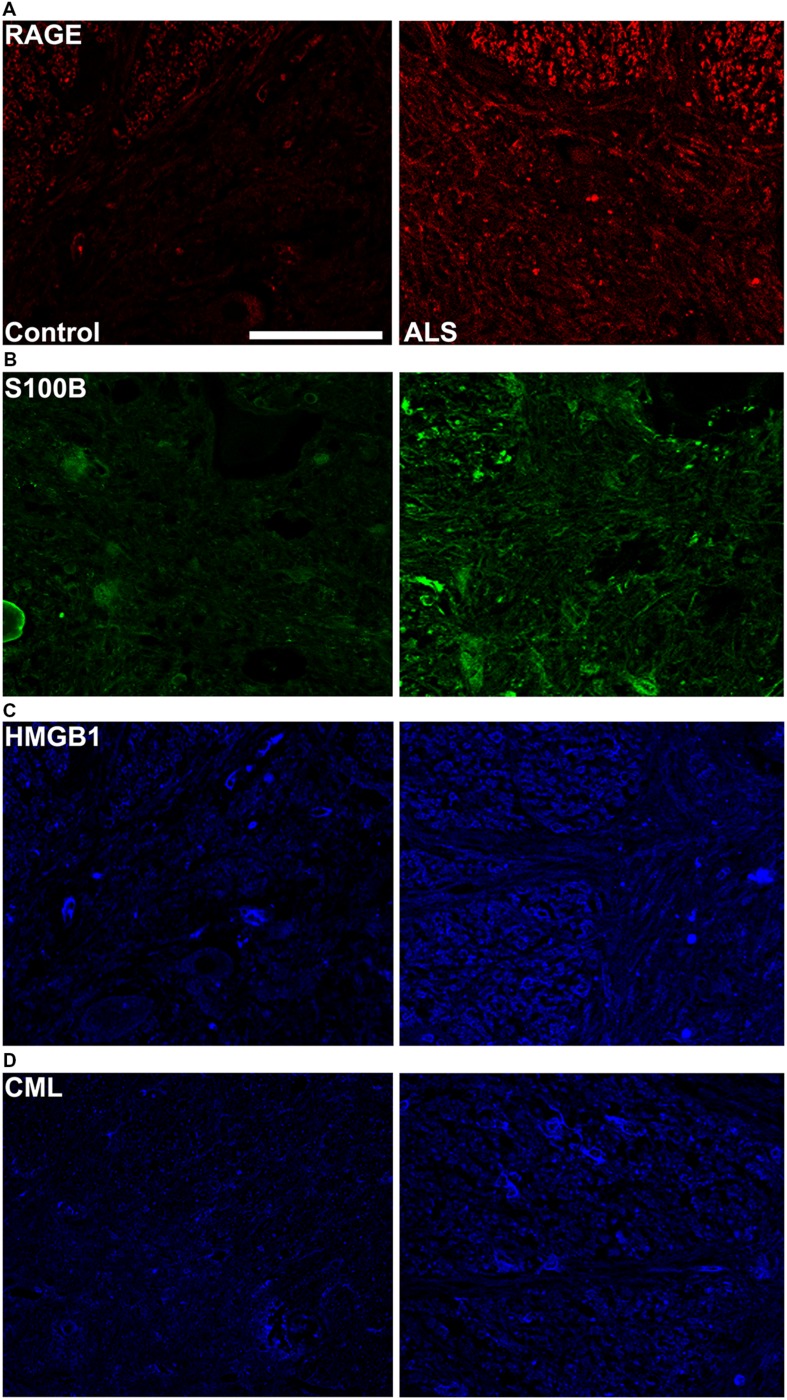
**High magnification images of immunostaining for RAGE and its ligands in the thoracic spinal cord.** Increased immunostaining pattern on the border of gray (lamina IX) and white matter was observed for **(A)** RAGE, **(B)** S100B, **(C)** HMGB1 and **(D)** CML in ALS versus control samples. Scale bar: 50 μm.

Here, for the first time, we aimed to establish the expression of RAGE and RAGE ligands in control human spinal cord and to determine whether and to what extent these levels were upregulated in ALS tissue. We demonstrate by immunohistochemistry a significantly increased expression of RAGE in human ALS spinal cord versus control samples. In the control thoracic spinal cord, overall RAGE expression was low as compared to ALS samples, (**Figures 1A,D**, **2A**, and **3A**). Quantification of RAGE immunofluorescence intensity was significantly higher in ALS tissue versus control (**Figure 1B**). By quantitative real-time PCR (RT-PCR), we found a trend for increased mRNA expression for *AGER*, the gene encoding RAGE, in thoracic spinal cords from ALS subjects versus controls (**Figure 1C**).

**FIGURE 4:**
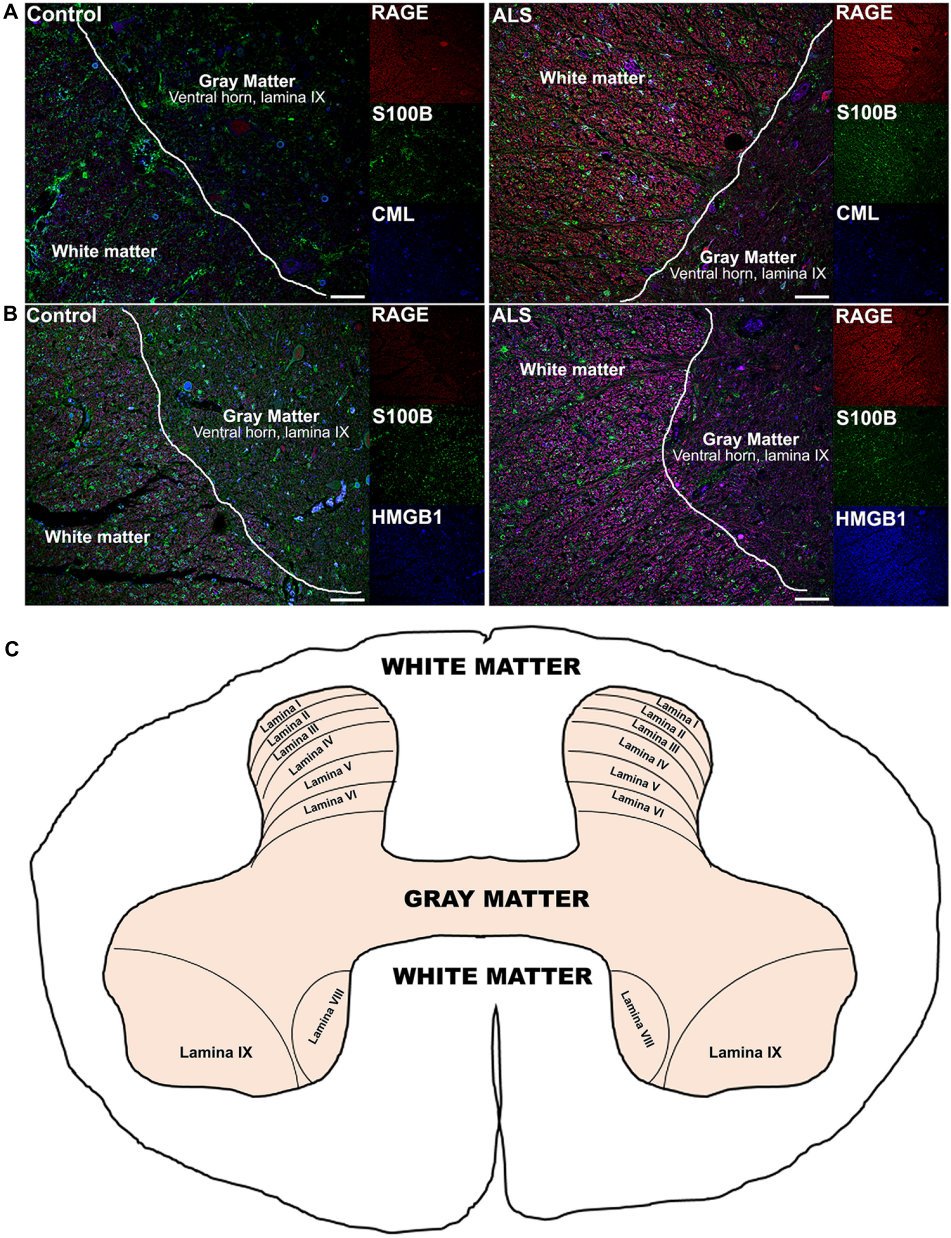
**Co-expression of RAGE and RAGE ligands S100B, CML, and HMGB1 is higher in human ALS spinal cord. (A)** Triple staining for RAGE (red), S100B (green), CML (blue) revealed increased immunoexpression of these proteins in the ALS spinal cord (**A**, right) as compared to controls (**A**, left) and a high degree of RAGE/ligand overlapping was observed in ALS samples (merged images). **(B)** Expression of RAGE (red) and its ligands, S100B (green) and HMGB1 (blue) was highly increased in the ALS (**B**, right) spinal cord as compared to controls (**B**, left) and a high degree of RAGE/ligand co-expression observed in ALS samples (merged images); control (*n* = 6) vs. ALS samples (*n* = 5). Scale bar: 100 μm. **(C)** A schematic diagram showing different regions of spinal cord; for the purpose of the study we examined thoracic motor spinal cord ventral horn lamina IX and surrounding white matter.

**FIGURE 5:**
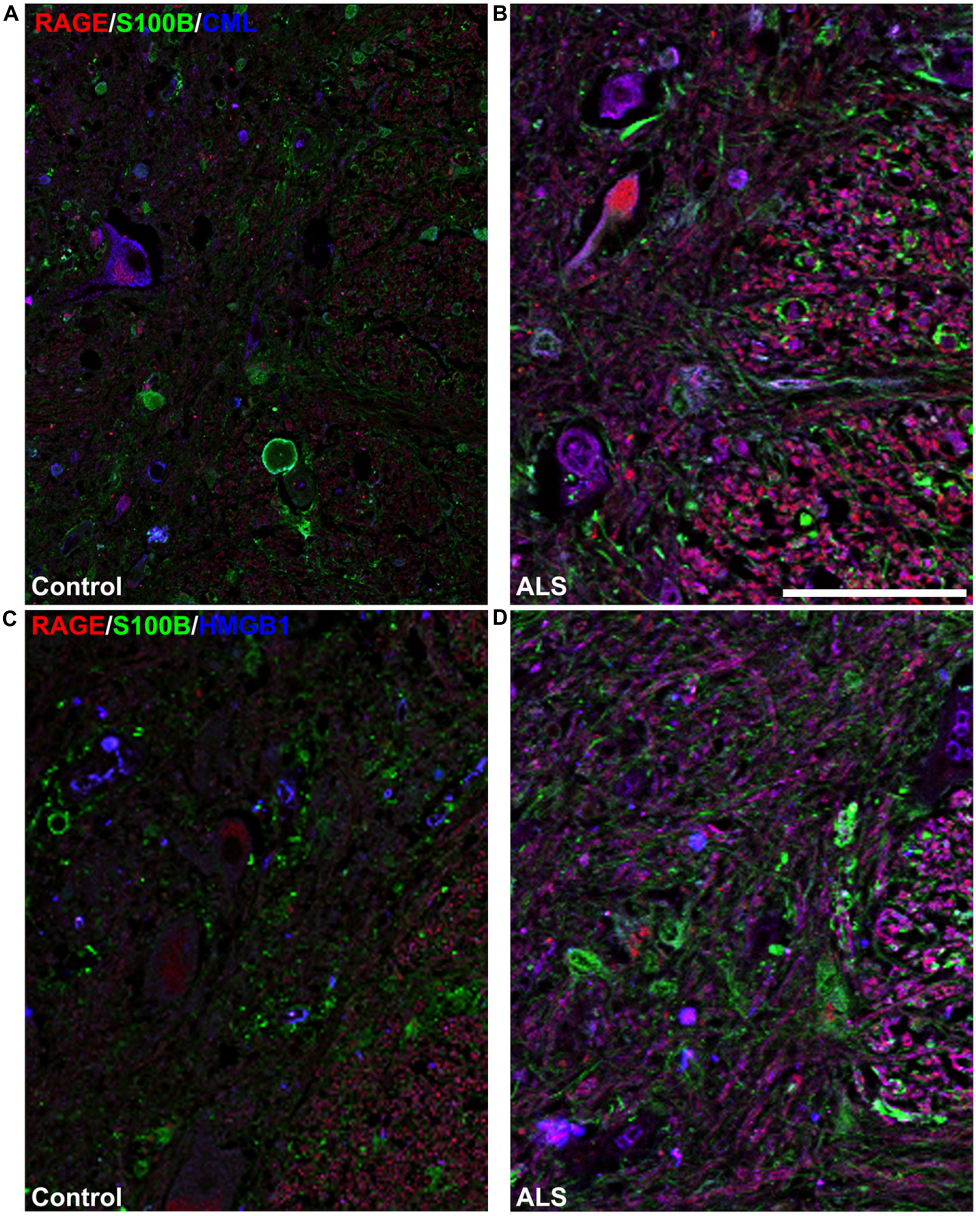
**High magnification images of white/gray matter showing triple staining for RAGE and its ligands S100B, CML, and HMGB1.** Immunostaining for RAGE (red) and its ligands S100B (green) and CML or HMGB1 (blue) revealed low immunoexpression in control tissue (**A** and **C**) and high immunoexpression in ALS tissue (**B** and **D**). Sections are representative of *n* = 6 control and *n* = 5 ALS tissue samples per condition. Scale bar: 100 μm.

Receptor for advanced glycation end-products ligands showed increased immunostaining in ALS thoracic spinal cord vs. control samples (**Figures 2B–D** and **3B–D**, **Figure 4C**) and analysis of immunofluorescence intensity was significantly higher in ALS tissue versus control for S100B, HMGB1 and CML, a prototype AGE protein (**Figures 2E–G**, respectively). S100B was sparsely distributed in the control tissue while in ALS thoracic spinal cord, S100B immunostaining was noticeably increased (**Figures 2B and 3B**). HMGB1 immunostaining was also highly increased in ALS thoracic spinal cord compared to control tissue (**Figures 2C and 3C**). CML immunoreactivity was sparsely distributed in control tissue while in ALS tissue, CML expression was increased (**Figures 2D and 3D**). By triple immunostaining, we show in control tissue, sparse overlapping of RAGE-S100B, moderate RAGE-CML and RAGE-HMGB1 immunostaining (**Figures 4A,B and 5A,C**) whereas in ALS tissue, we found a high degree of overlapping expression patterns for all ligands with RAGE (**Figures 4A,B and 5B,D**).

**FIGURE 6:**
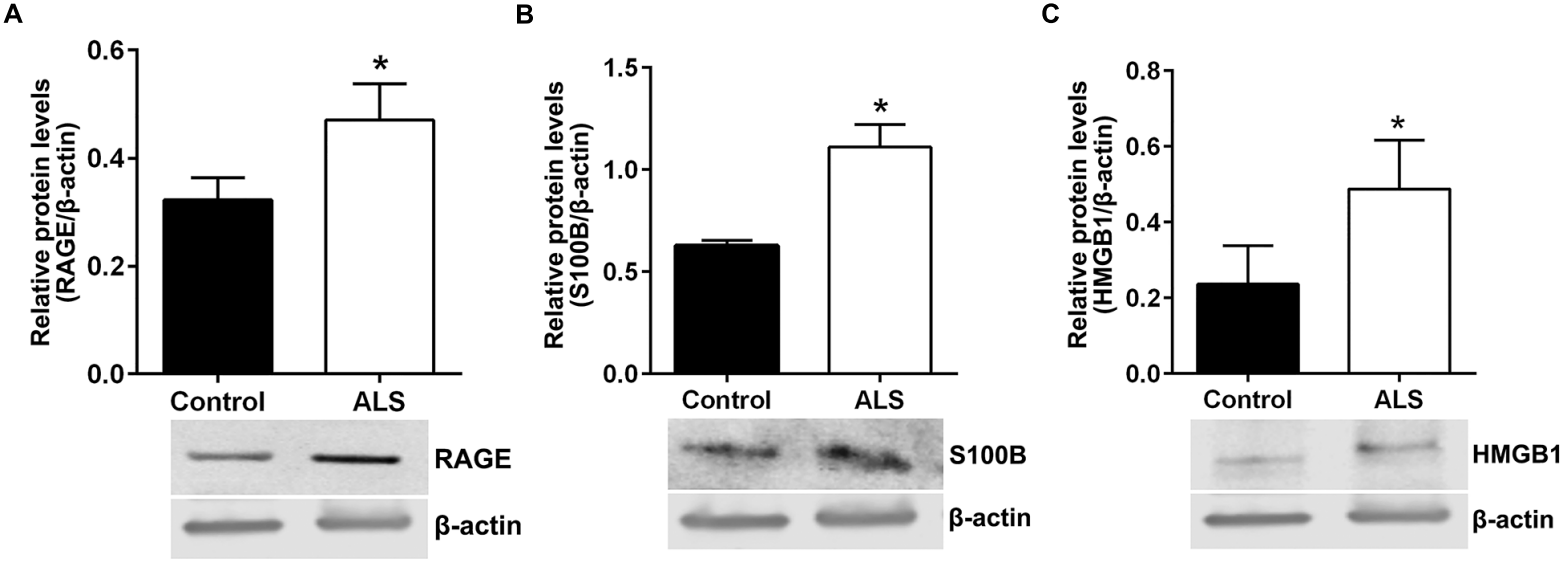
**Protein levels of RAGE and its ligands S100B and HMGB1 are higher in human ALS spinal cord.** Western blot analysis of RAGE **(A)**, RAGE ligands S100B **(B)** and HMGB1 **(C)** in control and ALS spinal cord tissue. The original blots were stripped followed by incubation with the other antigens under study. Signal for test antigen was then normalized to β-actin and the relative band densities were reported. *n* = 3 subjects/group. Error bars represent mean ± SEM, ^∗^*p* < 0.05.

Next, we sought to determine relative protein levels of RAGE and RAGE ligands in thoracic spinal cord samples from ALS subjects and age-matched controls. Western blotting revealed that ALS spinal cord tissue had significantly higher protein levels of RAGE, S100B, and HMGB1 compared to control samples (**Figures 6A–C**, respectively).

## Discussion

In summary, we demonstrate by immunohistochemistry, quantitative RT-PCR and Western blotting, that ALS spinal cord tissue displays increased expression of RAGE and RAGE ligands S100B, CML and HMGB1, suggesting a possible role for the RAGE pathway in ALS. Until the present time, data on RAGE expression in ALS patients has been limited to qualitative rather than quantitative analysis of RAGE expression in ALS patient spinal cord sections (Casula et al., 2011). Casula et al. (2011) reported that there was no significant change in *AGER* mRNA expression in ALS spinal cord, however, our study revealed a significant increase in RAGE expression in ALS spinal cord by immunohistochemistry, quantitative RT-PCR and Western blotting. It has been shown that on average, the correlation between mRNA and protein level is estimated at 40% and since mRNA transcripts are produced at much lower rates than proteins in mammalian cells (Vogel and Marcotte, 2012), it is likely that the differences in mRNA versus protein levels of RAGE contribute to the observed discrepancy. A distinct study focused on quantification of circulating soluble endogenous RAGE (sRAGE), a putative natural antagonist of RAGE in the plasma of control and ALS patients. This study revealed that sRAGE levels were lower in the ALS patients and suggested a link between low sRAGE levels and accelerated neurodegeneration in ALS (Ilzecka, 2009). It is plausible that the higher expression of RAGE ligands in the ALS subjects might reduce available sRAGE for immunoreactivity in ELISA studies. Alternatively, it is possible that subjects with inherently lower sRAGE levels were more vulnerable to ALS in the setting of other predisposing factors, such as genetic vulnerability. Given that sRAGE is a natural scavenger of RAGE ligands, it is interesting to speculate on the effects of sRAGE administration as a therapeutic for ALS treatment, as it has been proposed for metabolic and inflammatory diseases (Schmidt, 2015).

Enhanced RAGE expression has been observed in other neurodegenerative disorders, such as AD (Sasaki et al., 2001; Wang et al., 2009) and Huntington’s disease (Ma and Nicholson, 2004; Anzilotti et al., 2012), thereby suggesting a potential role for RAGE in the pathogenesis of these diseases. Indeed, a specific polymorphism of the *AGER* gene (G82S) has been associated with increased risk of neurodegeneration in two distinct populations of Chinese and European descent in AD (Daborg et al., 2010; Li et al., 2010). No studies to date, to our knowledge, have examined the known polymorphisms of *AGER* in ALS.

The observed increase in expression of RAGE binding AGEs, such as CML, reported previously in the spinal cord of ALS patients (Shibata et al., 1999, 2000; Kikuchi et al., 2002), further implicates AGE-RAGE contribution to the ALS pathogenesis, potentially via neuroinflammation and oxidative stress mediation of neuronal damage. Studies show that RAGE may activate macrophage and monocyte inflammatory responses and perpetuate inflammation in neurodegenerative diseases, (Grillo and Colombatto, 2008; Yan et al., 2009; Juranek et al., 2013b), likely exacerbating neuronal damage in ALS spinal cord.

HMGB1, another RAGE interacting partner and a member of the family of damage associated molecular pattern molecules (Tang et al., 2011), has been implicated in the pathogenesis of a number of neurodegenerative disorders such as Parkinson’s disease (Gao et al., 2011), AD (Takata et al., 2004, 2012), multiple sclerosis and ALS (Lo Coco et al., 2007; Andersson et al., 2008; Casula et al., 2011; Mazarati et al., 2011) via inflammatory toll like receptor/RAGE signaling pathways activation. It has been shown that increased expression of HMGB1 correlates with elevated levels of RAGE and contributes to memory impairment (Mazarati et al., 2011), demyelination (Andersson et al., 2008), and neurodegeneration (Gao et al., 2011). The results of our study show that HMGB1 is significantly increased and highly co-expressed with RAGE in human ALS spinal cord; further studies will be required to determine if HMGB1 plays important roles in neurodegenerative processes in the ALS spinal cord.

Similar to HMGB1, elevated levels of astrocyte S100B, another of RAGE ligands, have been observed in serum of ALS patients (Sussmuth et al., 2010) and in rat motor neurons exposed to cerebrospinal fluid from ALS patients (Shobha et al., 2010). Further, increased cerebrospinal fluid levels of S100B have been also reported in Parkinson’s disease (Sathe et al., 2012), AD (Edwards and Robinson, 2006), and schizophrenia (Schmitt et al., 2005), implying roles for S100B in the pathogenesis of neurodegenerative diseases. Reports show that RAGE-mediated inflammatory responses trigger S100B activated microglia stimulation in the brain (Bianchi et al., 2011), leading to neuronal damage and neurodegeneration and resulting in symptomatic brain disorders. In addition to potential links to inflammation, studies show that in the cerebellum of spinocerebellar ataxia type 1 mouse model, S100B-RAGE interaction leads to increased oxidative stress and further damages neurons contributing to the progression of the disease (Hearst et al., 2011). Our study reveals increased expression of S100B in human and mouse ALS spinal cord indicating that S100B may play an important role in the pathogenesis of the disease; it remains to be determined whether S100B-RAGE interactions in the spinal cord mediate damage via inflammation, oxidative stress or other distinct mechanisms.

**FIGURE 7:**
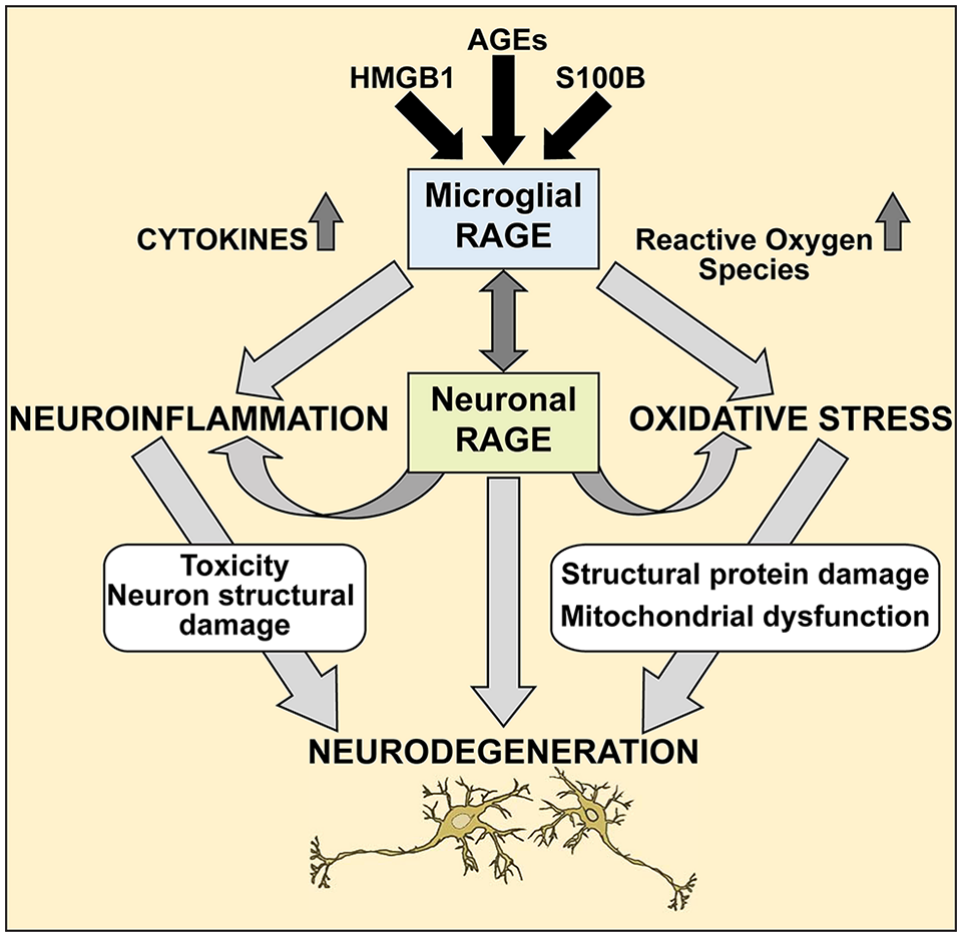
**A proposed mechanism of RAGE action in ALS spinal cord.** We propose that during pathological processes in ALS, neuronal and microglial RAGE becomes activated by RAGE ligands such as AGEs, S100B, and HMGB1. Once activated, RAGE triggers a cascade of metabolic changes, contributing to the release of reactive oxygen species (ROS) and inflammatory cytokines, subsequently resulting in altered protein structures and misfolded protein accumulation, impaired mitochondrial function and growing energy deficits ultimately leading to neuronal dysfunction and apoptosis.

Further studies are needed to elucidate the precise molecular mechanisms of the RAGE/AGE axis in ALS, however our studies provide a molecular glimpse into these processes. It is reasonable to speculate that selective loss of motor neurons in ALS occurs via the RAGE/AGE pathway (**Figure 7**). Mounting evidence indicates activated microglia to be a hallmark feature of many neurodegenerative diseases, including ALS disease progression (Hall et al., 1998; Henkel et al., 2004; Boillee et al., 2006). RAGE and its ligands have been implicated in mediating microglia activation, resulting in chronic inflammation and oxidative stress which upregulates RAGE and ligands such as S100B and HMGB1, leading to increased oxidative stress and thus creating a positive feedback loop that most likely results in neuronal cell death (Yan et al., 1996; Lue et al., 2001; Bianchi et al., 2010, 2007; Fang et al., 2010). Furthermore, in ALS microglial cells, upregulation of the RAGE/AGE molecules induces formation of reactive oxygen species (ROS; Frakes et al., 2014) and recently these toxic species were shown to elicit specific motor neuron cell death in a non-cell-autonomous model of ALS via the c-Abl pathway (Rojas et al., 2015).

Whether RAGE and its ligands are the cause or the modifier of neurodegenerative disease has yet to be elucidated. However, the upregulation of these molecules may indicate a mechanism, a biomarker or both. Our findings link upregulation of RAGE and its ligands in ALS affected tissue indicating that these molecules are worthy of further investigation.

In summary, our study is the first to report on differential, increased expression of inflammatory RAGE and its ligands in human spinal cord affected by ALS. It must be noted, however, that our study has been limited to the final stage of the disease due to the timing of availability of spinal cord tissues consequent to patients’ death and limited sample numbers. No earlier information on RAGE and its ligand expression could be obtained for comparative purposes from subjects with ALS expiring prior to the end stage of the disease. Nevertheless, we propose that our study provides molecular insights into ALS pathogenesis and suggests that further probing of the RAGE hypothesis as a mediator of pathogenesis in human ALS is rational.

## Author Contributions

JJ and GD wrote the manuscript, performed, and analyzed experiments. JW performed experiments. DL and JK collected and provided samples for the study. AMS wrote the manuscript and analyzed data.

## Conflict of Interest Statement

The authors declare that the research was conducted in the absence of any commercial or financial relationships that could be construed as a potential conflict of interest.

## Abbreviations

AGEs: advanced glycation end products
ALS: Amyotrophic lateral sclerosis
CML: carboxy methyl lysine
HMGB1: High Mobility Group Box-1
RAGE: receptor for advanced glycation end products
